# Correlates of Sedentary Time and Physical Activity Among Preschool-Aged Children

**Published:** 2011-10-15

**Authors:** Diana H. Dolinsky, Rebecca J. Namenek Brouwer, Truls Østbye, Kelly R. Evenson, Anna Maria Siega-Riz

**Affiliations:** Duke University Medical Center, Durham, North Carolina; Duke University Medical Center, Durham, North Carolina; Duke University Medical Center, Durham, North Carolina; Gillings School of Global Public Health, University of North Carolina at Chapel Hill; Gillings School of Global Public Health and Carolina Population Center, University of North Carolina at Chapel Hill, and Carolina Population Center, University of North Carolina at Chapel Hill

## Abstract

**Introduction:**

Few studies have examined the correlates of objectively measured amounts of sedentary time and physical activity in young children. We evaluated the demographic, biological, behavioral, social, and environmental correlates of the amount of sedentary time and moderate-to-vigorous physical activity (MVPA) as measured by accelerometry in preschool-aged children.

**Methods:**

We obtained baseline measurements of physical activity by using an Actical accelerometer among 337 preschool-aged children (aged 2-5) of overweight or obese mothers. For children, we defined sedentary time as less than 12 counts per 15 seconds and MVPA as 715 or more counts per 15 seconds. Body mass index of the mother and child (calculated from measured height and weight) and maternal physical activity as measured by accelerometer were included as potential correlates. Mothers self-reported all other potential correlates. We used multivariable linear regression analyses to examine correlates of the amount of sedentary time and MVPA.

**Results:**

Children had an average of 6.1 hours per day of sedentary time and 14.9 minutes per day of MVPA. In multivariable analysis, boys (*P* <.001) had fewer minutes per day of sedentary time, whereas older children (*P* <.001), boys (*P* <.001), children in high-income households (>$60,000/y [*P* = .005]), and children who spent more time outdoors (*P* = .001) had more MVPA.

**Conclusion:**

Both modifiable and nonmodifiable factors were correlated with preschool children's amount of MVPA, which can be helpful when designing interventions for this age group. The lack of correlates for sedentary time indicates the need for further investigation into this behavior.

## Introduction

Approximately one-fifth of preschool-aged children are overweight, and this prevalence increases as children age (1). Longitudinal studies indicate that children's amount of physical activity is inversely related to subsequent increases in adiposity, and the amount of sedentary time is directly related to increases in adiposity (2-4). Recent studies suggest that preschool-aged children are often inactive, spending less than 5% of their day in moderate-to-vigorous physical activity (MVPA) and more than three-fourths of their waking hours in sedentary pursuits (5,6).

Early interventions are necessary to alter these behaviors. However, even a basic understanding of the correlates of these behaviors is lacking. Although studies in older children have assessed the correlates of physical activity and of being sedentary (7,8), few studies examine this in preschool-aged children (9,10). The evidence examining correlates using objective measures is especially lacking (9,10). Reviews that have assessed correlates of physical activity and sedentary time in children have classified correlates into groups, such as demographic and biological; psychological, cognitive, and emotional; behavioral attributes and skills; social and cultural; and physical environment domains (8,11). Most of the studies in preschoolers assess correlates in only 1 or 2 of these domains, and few consider objectively measured maternal physical activity as a potential correlate (9,10). Further studies that use objective measures in preschool-aged children are needed to investigate the correlates of these behaviors more comprehensively across multiple domains. We used baseline data from the Kids and Adults Now! Defeat Obesity (KAN-DO) Study to examine potential demographic, biological, behavioral, social, and environmental correlates of objectively measured amounts of sedentary time and MVPA in a sample of children aged 2 to 5 years.

## Methods

### The KAN-DO Study participants

We used baseline data obtained from September 2007 through November 2009 from KAN-DO, a randomized, controlled intervention trial of 400 overweight postpartum mothers and their preschool-aged children. Details of the KAN-DO Study are published elsewhere (12). Briefly, inclusion criteria for the KAN-DO Study included a postpartum maternal body mass index (BMI) of at least 25 kg/m^2^, delivery of an infant 1 to 7 months before randomization, and a child aged 2 to 5 years in the household. Exclusion criteria included mother less than 18 years old, mother unable to speak and read English, no regular access to a telephone, lack of a permanent mailing address, and presence of a health condition precluding daily physical activity in the mother or child. If multiple children in the home were aged 2 to 5 years, we chose the child born in the earliest month of the year for study participation.

We recruited study participants from 14 counties in the Triangle and Triad areas of North Carolina by using a combination of birth certificate records, publicly available mailing lists, and flyers posted in clinic and community locations. Of more than 40,000 women who received information about the KAN-DO Study, 4,445 were screened; and of those, 1,617 refused and 2,180 were ineligible, leaving 648 who were eligible and interested. Of those, 152 did not attend their first scheduled appointment, 80 refused or were deemed ineligible at their first study appointment, and 16 did not complete all components of the baseline assessment. This left 400 eligible mothers from whom we obtained written informed consent. The participants were randomized based on 4 strata (child's age, mother's race, site, and days from birth of recent child). The institutional review boards of Duke University Medical Center and the University of North Carolina at Greensboro approved this study.

### Outcomes

The outcomes were measured objectively by using an accelerometer. Accelerometers are small instruments worn on the body that measure accelerations that can be converted to intensity of physical activity. The measurements are averaged over prespecified time periods called epochs (13). In the KAN-DO Study at baseline, children were instructed to wear an Actical omnidirectional accelerometer (Mini Mitter Co, Inc, Bend, Oregon) placed at the hip for 7 days. They were instructed to remove the monitor only for bathing and nighttime sleeping. The accelerometers were water-resistant, and children were instructed to also wear them for water-based activity. The accelerometers were set to record activity in 15-second epochs. The 2 outcomes of interest were child's minutes per day of sedentary time and child's minutes per day of MVPA. Sedentary time was defined by using a cutpoint of less than 12 counts per 15 seconds (14). MVPA was defined by using a cutpoint of 715 counts or more per 15 seconds (15). To be included in the analysis, children needed at least 3 valid days of wear (2 weekdays and 1 weekend day with at least 6 h/d of wear). Accelerometer data were available for 392 children; 55 of these children had fewer than 3 valid days of wear, resulting in 337 children available for analysis.

### Potential correlates

The correlates (Table 1) were divided into demographic, biological, behavioral attributes and skills, social and cultural, and physical environment domains adapting a framework used by Sallis et al (8). The mothers were asked to wear the same type of accelerometer as the child, but set to 60-second epochs, at the hip for 7 days. They were instructed to remove them only for bathing or nighttime sleeping and to wear them for water-based activity. Maternal physical activity was examined as total counts per day. For the maternal accelerometer data to be included in the analysis, mothers also had to have at least 3 valid days (2 weekdays and 1 weekend day with at least 6 h/d) of wear. For both the mother and child, we measured height by using the Seca 214 portable stadiometer (Seca, Hamburg, Germany) and measured weight by using the Tanita BWB-800s digital scale (Tanita Corp of America, Inc, Tokyo, Japan). We categorized children as underweight, healthy weight, overweight, or obese by using American Academy of Pediatrics recommendations (16). All other potential correlates were from the baseline questionnaires completed by the mother. To screen for postpartum depression, we used the Edinburgh Postnatal Depression Scale (EPDS), a 10-item questionnaire with a maximum score of 30, and we considered scores of 13 or higher as a positive screen for depression (17). To assess the presence of a chronic health problem among the mothers, we asked, "Do you have any longstanding illness, disability, or medical condition? That is, anything that affects your work or other regular daily activities such as type 2 diabetes, cancer, and heart disease?"

### Statistical analysis

The outcomes were modeled by using a natural log transformation to meet the assumptions of linear regression. Since the study was conducted at 2 study sites (Durham and Greensboro), study site was included in all models to account for potential site difference. To account for differences in wear time, we also included mean hours per day of monitoring for the children in all models. We first conducted linear regression between each correlate and the transformed outcome to produce minimally adjusted β coefficients, adjusted only for site and wear time. We then conducted multivariable analysis to produce adjusted β coefficients. Because the outcome was log transformed, the β coefficients were retransformed ([exponentiated β coefficient − 1] × 100) to represent the percentage of change in the outcome per unit change in each independent variable. We explored collinearity between correlates using criteria of a condition index of 30 or higher to consider the presence of multicollinearity (18). Linearity with the outcome for each potential correlate was explored and, if necessary, correlates were either transformed or modeled by using indicator variables. Maternal physical activity was modeled by using quartiles, with the highest quartile representing the most total counts per day of maternal activity.

We created separate models for each of the domains of the potential correlates (demographic, biological, behavioral attributes and skills, social and cultural, and physical environment). For each of these models, we conducted a partial *F* test for the potential correlates only because site and wear time were kept in all models. If the *P* value was <.20, we used backward selection with partial *F* tests to remove variables in the domain with *P* values of ≥.20. Variables retained in these separate models were combined in the full model. To create the final model, we removed each domain from the full model with partial *F* tests using a *P* value ≥.20 as the criteria for removal. We considered variables with a *P* value of ≤.05 to be significant. We ensured that the final models met the assumptions of linear regression. For the model examining the outcome of sedentary time, we removed 8 observations from the multivariable model because they led to violations of the assumptions of linear regression in the final model. For the model examining MVPA, we removed 1 observation from the multivariable model for the same reason. These observations were removed because they had studentized residuals of greater than 3 or less than −3 in the final models. For consistency, these observations were also removed from the unadjusted analyses. We performed all analyses with Stata 11.0 (StataCorp LP, College Station, Texas).

## Results

### Characteristics of the analysis sample

**Figure 1.**
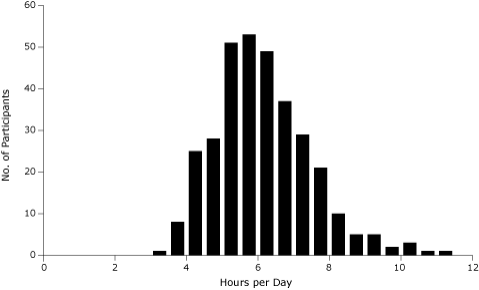
Child's amount of sedentary time, KAN-DO Study, North Carolina, 2007-2009 (n = 329). Children's mean (SD) amount of sedentary time was 6.1 (1.3) hours per day. Abbreviation: KAN-DO (Kids and Adults Now! Defeat Obesity).

**Figure 2.**
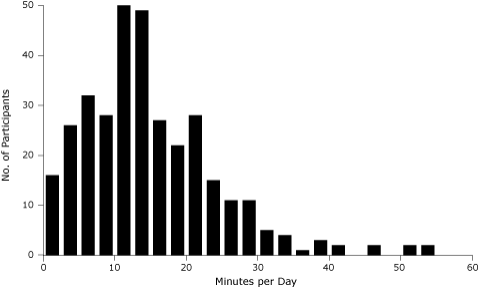
Child's amount of moderate-to-vigorous physical activity, KAN-DO Study, North Carolina, 2007-2009 (n = 336). Children's mean (SD) amount of moderate-to-vigorous physical activity was 14.9 (9.5) minutes per day. Abbreviation: KAN-DO, Kids and Adults Now! Defeat Obesity.

The 337 children in the analysis sample (Table 1) had a mean age of 3.5 years (standard deviation [SD], 1.1); 58% were boys. The children were monitored for a mean (SD) of 6.3 (1.4) days and had 6.1 (1.3) hours per day of sedentary time (Figure 1), and 14.9 (9.5) minutes per day of MVPA (Figure 2). Of the participants, 61% of children were seen at the Durham study site and 39% at the Greensboro study site. The variable with the most missing information was presence of a chronic health problem in the mother (12% missing). All other variables had less than 3% of observations missing.

**Table:** 

**Hours per Day **	**Number of Participants**
0 to <0.5	0
0.5 to <1.0	0
1.0 to <1.5	0
1.5 to <2.0	0
2.0 to <2.5	0
2.5 to <3.0	0
3.0 to <3.5	1
3.5 to <4.0	8
4.0 to <4.5	25
4.5 to <5.0	28
5.0 to <5.5	51
5.5 to <6.0	53
6.0 to <6.5	49
6.5 to <7.0	37
7.0 to <7.5	29
7.5 to <8.0	21
8.0 to <8.5	10
8.5 to <9.0	5
9.0 to <9.5	5
9.5 to <10.0	2
10.0 to <10.5	3
10.5 to <11.0	1
11.0 to <11.5	1
11.5 to <12.0	0

**Table:** 

**Minutes per Day **	**Number of Participants**
0 to <2.5	16
2.5 to <5.0	26
5.0 to <7.5	32
7.5 to <10.0	28
10.0 to <12.5	50
12.5 to <15.0	49
15.0 to <17.5	27
17.5 to <20.0	22
20.0 to <22.5	28
22.5 to <25.0	15
25.0 to <27.5	11
27.5 to <30.0	11
30.0 to <32.5	5
32.5 to <35.0	4
35.0 to <37.5	1
37.5 to <40.0	3
40.0 to <42.5	2
42.5 to <45.0	0
45.0 to <47.5	2
47.5 to <50.0	0
50.0 to <52.5	2
52.5 to <55.0	2
55.0 to <57.5	0
57.5 to 60.0	0

The 337 children in the analysis sample were not different from the 63 children enrolled in the KAN-DO Study without sufficient accelerometry data for inclusion in our analysis with respect to maternal age, maternal marital status, maternal education, and maternal ethnicity. The 337 children in the analysis sample were on average 0.5 years older than the 63 children not included (*P *< .001), more likely to be boys (58% included vs 44% not included; *P *< .05), and more likely to have a household income greater than $60,000 per year (59% included vs 45% not included, *P* < .05). Maternal race differed among the children in the analysis sample (77% white, 23% nonwhite), compared with those not included (63% white, 37% nonwhite; *P* = .02).

### Comparison of potential correlates with child's amount of sedentary time

In minimally adjusted analyses (adjusting only for site and monitoring time), correlates of the child's amount of sedentary time were child's sex, child's time spent outdoors, and maternal activity (Table 2). In multivariable analyses, the final model contained only demographic, biological, and social and cultural variables. The only correlate of sedentary time in the final model was child's sex. Boys had less sedentary time than girls (*P* < .001).

### Comparison of potential correlates with child's amount of MVPA

In minimally adjusted analyses, correlates of the child's amount of MVPA were child's age, child's sex, household income, maternal education, and mother's report of child's time spent outdoors. The final model contained only demographic and physical environment variables. Correlates of MVPA were child's sex, child's age, household income, and child's time spent outdoors. Both child's age and child's time spent outdoors were modeled with squared terms because they exhibited quadratic relationships with the outcome. Older children engaged in more MVPA than younger children (*P* = <.001), and boys had more MVPA than girls (*P* < .001). Children who spent more time outdoors had more MVPA (*P* = .001). Children in households with a household income of at least $60,000 per year had more MVPA than children in households with a lower income (*P* = .005).

## Discussion

In our sample, 1 demographic factor, sex, was a correlate of children's amount of sedentary time, and several demographic and physical environment factors were correlates of children's amount of MVPA. Variables in the biological, social and cultural, and behavioral attributes and skills domains were not correlates of either sedentary time or MVPA in our sample.

The small amount of MVPA and large amount of sedentary time in our sample of young children is of concern but is not uncommon. Taylor et al (who also used the Actical accelerometer) found similar results (19). Although 3-year-old children in that study had more MVPA than our sample, the mean minutes per day of MVPA in children aged 4 to 5 years was 16 minutes per day to 23 minutes per day, which is comparable to our findings (19). Other studies using different accelerometers for objective measurements have found that young children spend approximately three-fourths of their waking hours sedentary (5,6).

To our knowledge, ours is the first study to evaluate the relationship between overall sedentary time in preschool-aged children and maternal physical activity as measured by accelerometry. We found no relationship between maternal overall physical activity and children's sedentary time. We did identify an association between child's sex and child's amount of sedentary time. Other studies investigating the relationship between child's sex and sedentary time have found inconsistent results (20-22). We found no other correlates of sedentary time in preschool-aged children. Hinkley et al, in a review article, concluded that there is a lack of consistent evidence of a relationship between amount of sedentary time and other potential correlates in preschool-aged children (10).

In comparison with studies investigating sedentary time, more studies have investigated correlates of the amount of MVPA as measured by accelerometry in this age group. Most studies have shown that boys engage in more MVPA than girls (20,23-26), which is in agreement with our findings. Children spending more time outdoors had more physical activity in our sample, which is in agreement with 1 other study (23). We found a positive association between household income and child's amount of MVPA. Our findings contrast with those of other studies in this age group that found no difference; however, these other studies were conducted outside the United States where the relationship between income and child's activity may differ (24,27). Perhaps the association between household income and MVPA represents differences in neighborhood physical activity options for families of different socioeconomic status. Studies in older children have found that lower socioeconomic status is associated with a lower availability of physical activity facilities and a lower subsequent physical activity level (28).

In addition, we found that older preschool-age children engaged in more MVPA than younger ones. Other studies using accelerometry have found varied results regarding the association between the child's age and amount of MVPA in preschoolers. Some have found no association (20), but others found that MVPA is higher in older children (25,26,29), and in contrast, others have found that MVPA is lower in older children (19). In our study, maternal physical activity was not a correlate of child's MVPA. Another study using accelerometry found that parental physical activity as measured by accelerometry was related to the child's, but in that study, parental activity included the activity of mothers or fathers, or both (29).

The main strengths of our study are that we obtained information on a relatively large number of children and evaluated various potential correlates over numerous domains, including measurements of maternal physical activity. In addition, we used objective measures of our outcomes. Specifically, the Actical accelerometer is omnidirectional; it assesses activity in many unspecified dimensions, whereas most previous studies used other accelerometers that assess activity in only 1 to 3 prespecified axes and, theoretically, may not capture preschooler activity as well (13).

Limitations to our study include not obtaining measures of other potential influences on the child's behavior, such as paternal physical activity and the neighborhood environment. Also, we measured total sedentary time, and the correlates of different types of sedentary behaviors (television viewing vs reading) may be different for these activities. Inclusion criteria for the study included that the mother must be overweight or obese, and the correlates of children's behavior may differ if they have a normal weight or underweight mother. In addition, participants in our study were more educated and had a higher household income than the North Carolina population, as described using 2000 US Census information (30). Our results may have limited generalizability because of these issues.

In summary, only 1 nonmodifiable (sex) correlate was identified for sedentary time, and both nonmodifiable (child's age, sex) and modifiable (household income, child's time spent outdoors) correlates were identified for MVPA in preschool-aged children. Knowledge of these correlates may be helpful in designing and targeting interventions to decrease the amount of sedentary time and increase the amount of MVPA in young children.

## Figures and Tables

**Table 1:** Descriptive Characteristics of Analysis Sample, KAN-DO Study, North Carolina, 2007-2009 (n = 337)

Characteristic	n^a^	Mean (SD) or %

Demographic		
Child's age, y	337	3.5 (1.1)
Maternal age, y	337	32.7 (4.9)
**Maternal race/ethnicity**		
White non-Hispanic	246	73%
White Hispanic	15	4%
Nonwhite	76	23%
**Child's sex**		
Boy	195	58%
Girl	142	42%
**Maternal marital status**		
Married	295	88%
Single/divorced/separated	29	9%
Living with partner	13	4%
**Annual household income, $**		
≤60,000	137	41%
>60,000	195	59%
**Maternal education**		
High school or less	41	12%
Some post-high school training or college	63	19%
College graduate or greater	233	69%
**Biological**		
**Child's body mass index category^b^**		
<5th percentile (underweight)	10	3%
≥5th to <85th percentile (healthy weight)	242	72%
≥85th to <95th percentile (overweight)	55	16%
≥95th percentile (obese)	30	9%
**Maternal body mass index, kg/m^2^**		
25 to <30	132	39%
30 to <35	111	33%
35 to <40	57	17%
≥40	37	11%
**Maternal depression screen^c^**		
Negative	278	82%
Positive	59	18%
**Maternal chronic health problem**		
Yes	29	10%
No	268	90%
**Behavioral attributes and skills**		
**Child's history of being breastfed in first 12 months**		
No breastfeeding	51	15%
Breast and formula fed	227	67%
Breastfed exclusively until 12 mo	59	18%
**Child's television watching, h/d**		
<2	187	55%
≥2	150	45%
**Child's sweetened beverage and soda consumption per day**		
None	157	47%
Some	180	53%
**Child sleeps too little**		
Usually or sometimes	81	24%
Rarely	253	76%
**Social and cultural**		
Maternal physical activity, counts/d^d^	330	113,037 (46,550)
**Maternal television watching, h/d**		
<2	145	43%
≥2	192	57%
**Maternal computer use, h/d**		
<2	261	77%
≥2	76	23%
**Physical environment**		
Child's time spent outdoors, h/d	330	2.0 (1.6)
**Childcare arrangement**		
Stay-at-home mother	176	52%
Some combination of child care that includes mother	79	23%
Full-time child care with someone other than mother	82	24%
**No. of children born to the mother**		
2	208	62%
3	82	24%
≥4	47	14%
**Opportunities indoors for gross motor play**		
Yes	297	88%
No	40	12%
**Opportunities outdoors for gross motor play**		
Yes	305	91%
No	32	10%
**Television in the child's bedroom**		
Yes	83	25%
No	254	75%

Abbreviations: KAN-DO, Kids and Adults Now! Defeat Obesity; SD, standard deviation.

a Numbers may not sum to totals because of missing numbers, and percentages may not sum to 100% because of rounding.

b Defined by using current American Academy of Pediatrics recommendations (16).

c Categorized by using the Edinburgh Postnatal Depression Scale, considering scores of 13 or greater as a positive screen for depression (17).

d Maternal physical activity is described in counts per day because cutoffs for MVPA and sedentary time were not available.

**Table 2:** Association of Potential Correlates With Child's Sedentary Time and Moderate-to-Vigorous Physical Activity (MVPA), KAN-DO Study, North Carolina, 2007-2009

Charcteristic	**Sedentary Time (n = 329)**				**MVPA (n = 336)**			

	**Minimally Adjusted^a^**		**Adjusted^b^**		**Minimally Adjusted^a^**		**Adjusted^b^**	

	%** Difference** (95% CI) **c**	***P*** Value	%** Difference** (95% CI) **c**	***P*** Value	%** Difference** (95% CI) **c**	***P*** Value	%** Difference** (95% CI) **c**	***P*** Value

Demographic								
**Child's age^d^**								
Linear term, y	−0.2 (−1.5 to 1.0)	.70	NA^e^	NC	218.1 (102.4 to 399.9)	<.001	187.1 (83.6 to 349.1)	<.001
Squared term, y^2^	NA	NC	NA	NC	−10.6 (−15.8 to −5.1)	<.001	−9.7 (−14.9 to −4.2)	.001
**Maternal age, y**								
<30	1 [Reference]	0.79	NA	NC	1 [Reference]	0.24	NA	NC
≥30	−0.4 (−3.4 to 2.7)		NA	NC	11.6 (−7.1 to 33.9)		NA	NC
**Maternal race/ethnicity**								
White non-Hispanic	1 [Reference]	0.23	NA	NC	1 [Reference]	0.35	NA	NC
White Hispanic	4.7 (−2.1 to 11.9)		NA	NC	−24.7 (−48.9 to 11.0)		NA	NC
Nonwhite	2.0 (−1.2 to 5.2)		NA	NC	−2.8 (−19.4 to 17.2)		NA	NC
**Child's sex**								
Boy	1 [Reference]	<.001	1 [Reference]	<.001	1 [Reference]	<.001	1 [Reference]	<.001
Girl	5.4 (2.8 to 8.2)		5.2 (2.3 to 8.2)		−25.6 (−36.2 to −13.4)		−24.0 (−33.5 to −13.0)	
**Maternal marital status**								
Married	1 [Reference]	.77	NA	NC	1 [Reference]	.51	NA	NC
Single/divorced/ separated	−0.9 (−5.4 to 3.8)		NA	NC	−7.3 (−29.8 to 22.6)		NA	NC
Living with partner	−2.1 (−8.4 to 4.6)		NA	NC	−19.6 (−46.1 to 19.9)		NA	NC
**Annual household income, $**								
≤60,000	1 [Reference]	.52	NA	NC	1 [Reference]	.02	1 [Reference]	.005
>60,000	0.9 (−1.8 to 3.7)		NA	NC	21.0 (3.2 to 41.9)		23.1 (6.4 to 42.3)	
**Maternal education**								
High school or less	1 [Reference]	.33	NA	NC	1 [Reference]	.04	NA	NC
Some post-high school training or college	3.6 (−1.2 to 8.6)		NA	NC	−11.2 (−33.1 to 17.9)		NA	NC
College graduate or greater	2.7 (−1.4 to 7.0)		NA	NC	14.5 (−10.1 to 45.8)		NA	NC
**Biological**								
**Child's body mass index category^f^**								
<5th percentile (underweight)	1 [Reference]	.32	NA	NC	1 [Reference]	.84	NA	NC
≥5th to <85th percentile (healthy weight)	0.9 (−6.5 to 8.9)		NA	NC	18.1 (−25.5 to 87.2)		NA	NC
≥85th to <95th percentile (overweight)	−2.4 (−10.1 to 5.9)		NA	NC	20.4 (−26.2 to 96.6)		NA	NC
≥95th percentile (obese)	0.2 (−8.1 to 9.2)		NA	NC	9.7 (−34.7 to 84.1)		NA	NC
**Maternal body mass index (kg/m^2^)**								
25 to <30	1 [Reference]	.13	1 [Reference]	.18	1 [Reference]	.37	NA	NC
30 to <35	−1.8 (−4.7 to 1.3)		−2.5 (−5.6 to 0.7)		−3.9 (−19.9 to 15.4)		NA	NC
35 to <40	2.9 (−0.9 to 6.8)		1.9 (−2.1 to 6.0)		−15.5 (−32.4 to 5.7)		NA	NC
≥40	0.6 (−3.7 to 5.2)		−1.1 (−5.8 to 3.8)		−15.5 (−35.0 to 9.8)		NA	NC
**Maternal depression screen^g^**								
Negative	1 [Reference]	.45	NA	NC	1 [Reference]	.22	NA	NC
Positive	−1.3 (−4.6 to 2.1)		NA	NC	−11.9 (−28.0 to 7.8)		NA	NC
**Maternal chronic health problem**								
Yes	1 [Reference]	.20	1 [Reference]	.15	1 [Reference]	.60	NA	NC
No	3.1 (−1.6 to 8.0)		3.5 (−1.2 to 8.5)		−6.9 (−28.8 to 21.9)		NA	NC
**Behavioral attributes and skills**								
**Child's history of being breastfed in first 12 months**								
No breastfeeding	1 [Reference]	.09	NA	NC	1 [Reference]	.36	NA	NC
Breast and formula fed	2.8 (−0.9 to 6.6)		NA	NC	−6.7 (−25.1 to 16.3)		NA	NC
Breastfed exclusively until 12 months	5.3 (0.6 to 10.2)		NA	NC	−17.4 (−37.1 to 8.5)		NA	NC
**Child's television watching (h/d)**								
<2	1 [Reference]	.20	NA	NC	1 [Reference]	.51	NA	NC
≥2	1.7 (−0.9 to 4.4)		NA	NC	5.4 (−9.8 to 23.3)		NA	NC
**Child's sweetened beverage and soda consumption per day**								
None	1 [Reference]	.69	NA	NC	1 [Reference]	.15	NA	NC
Some	−0.5 (−3.1 to 2.1)		NA	NC	12.3 (−4.0 to 31.4)		NA	NC
**Child sleeps too little**								
Usually or sometimes	1 [Reference]	.44	NA	NC	1 [Reference]	.28	NA	NC
Rarely	1.2 (−1.8 to 4.3)		NA	NC	−9.5 (−24.4 to 8.4)		NA	NC
**Social and cultural**								
**Maternal physical activity quartile**								
Lowest	1 [Reference]	.02	1 [Reference]	.13	1 [Reference]	.34	NA	NC
Second	5.3 (1.5 to 9.2)		3.7 (−0.3 to 7.9)		−14.7 (−31.7 to 6.5)		NA	NC
Third	0.7 (−3.0 to 4.5)		−0.4 (−4.2 to 3.6)		−15.4 (−32.3 to 5.6)		NA	NC
Highest	0.7 (−3.0 to 4.5)		−0.3 (−4.2 to 3.7)		−4.0 (−23.2 to 20.1)		NA	NC
**Maternal television watching (h/d)**								
<2	1 [Reference]	.53	NA	NC	1 [Reference]	.76	NA	NC
≥2	0.9 (−1.8 to 3.5)		NA	NC	−2.4 (−16.4 to 14.0)		NA	NC
**Maternal computer use (h/d)**								
<2	1 [Reference]	.68	NA	NC	1 [Reference]	.31	NA	NC
≥2	0.7 (−2.4 to 3.9)		NA	NC	−9.1 (−24.4 to 9.2)		NA	NC
**Physical environment**								
**Child's time spent outdoors (h/d)**								
**Categorical**								
<1	1 [Reference]	.005	NA	NC	NA	NC	NA	NC
≥1	−4.4 (−7.3 to −1.4)		NA	NC	NA	NC	NA	NC
**Continuous**								
Linear term (h/d)^e^	NA	NC	NA	NC	33.8 (18.7 to 50.7)	<.001	22.2 (9.6 to 36.2)	<.001
Squared term (h/d)^2^ e	NA	NC	NA	NC	−3.3 (−4.6 to −1.9)	<.001	−2.3 (−3.5 to −1.1)	<.001
**Child care arrangement**								
Stay-at-home mother	1 [Reference]	.43	NA	NC	1 [Reference]	.05	1 [Reference]	.98
Some combination of child care that includes mother	0.03 (−3.1 to 3.3)		NA	NC	22.7 (1.4 to 48.4)		−0.6 (−16.3 to 18.1)	
Full-time child care with someone other than mother	2.0 (−1.2 to 5.3)		NA	NC	19.4 (−1.1 to 44.1)		1.5 (−14.3 to 20.2)	
**No. of children born to the mother**								
2	1 [Reference]	.51	NA	NC	1 [Reference]	.55	NA	NC
3	1.8 (−1.3 to 5.0)		NA	NC	2.9 (−14.5 to 23.9)		NA	NC
≥4	0.2 (−3.7 to 4.2)		NA	NC	−10.6 (−29.0 to 12.7)		NA	NC
**Opportunities indoors for gross motor play**								
Yes	1 [Reference]	.11	NA	NC	1 [Reference]	.14	NA	NC
No	3.3 (−0.7 to 7.6)		NA	NC	−16.8 (−34.5 to 5.8)		NA	NC
**Opportunities outdoors for gross motor play**								
Yes	1 [Reference]	.34	NA	NC	1 [Reference]	.76	NA	NC
No	−2.1 (−6.3 to 2.3)		NA	NC	4.2 (−19.8 to 35.5)		NA	NC
**Television in the child's bedroom**								
Yes	1 [Reference]	.68	NA	NC	1 [Reference]	.60	NA	NC
No	−0.7 (−3.7 to 2.5)		NA	NC	−4.8 (−21.0 to 14.6)		NA	NC

Abbreviations: MVPA, moderate-to-vigorous physical activity; KAN-DO, Kids and Adults Now! Defeat Obesity; CI, confidence interval; NA, not applicable; NC, not calculated.

a All estimates are adjusted for site and child's mean hours per day of monitoring.

b Includes estimates for potential correlates remaining in the final model only.

c Because the outcome was log transformed, the estimate ([exponentiated β coefficient-1]×100) represents the percentage change in the outcome per unit change in each independent variable.

d Linearity with each potential correlate and the outcomes were examined. For age and both outcomes and child's time spent outdoors and MVPA, the potential correlates were most appropriately modeled by using transformation (including both linear and squared terms).

e NA indicates that the variable in the specified form was not evaluated in the model.

f Defined by using current American Academy of Pediatrics recommendations (16).

g Categorized by using the Edinburgh Postnatal Depression Scale, considering scores of 13 or higher as a positive screen for depression (17).
